# The Role of Hormones in the Differences in the Incidence of Breast Cancer between Mongolia and the United Kingdom

**DOI:** 10.1371/journal.pone.0114455

**Published:** 2014-12-23

**Authors:** Rebecca Troisi, Daavasambuu Ganmaa, Isabel dos Santos Silva, Dambadarjaa Davaalkham, Philip S. Rosenberg, Janet Rich-Edwards, Lindsay Frasier, Lauren Houghton, Craig Janes, Frank Stanczyk, Robert N. Hoover

**Affiliations:** 1 Epidemiology and Biostatistics Program, Division of Cancer Epidemiology and Genetics, National Cancer Institute, National Institutes of Health, Department of Health and Human Services, Rockville, Maryland, 20850, United States of America; 2 Departments of Nutrition and Epidemiology, Harvard School of Public Health, Boston, Massachusetts, 02115, United States of America; 3 Health Sciences University of Mongolia, Ulaanbaatar, Mongolia; 4 Department of Non-communicable Disease Epidemiology, London School of Hygiene and Tropical Medicine, London, WC1E 7HT, England, United Kingdom; 5 Channing Laboratory, Department of Medicine, and Connors Center for Women's Health and Gender Biology, Brigham and Women's Hospital, Harvard Medical School, Boston, Massachusetts, 02115, United States of America; 6 Department of Pediatric Oncology, Dana Farber Cancer Institute, Boston, Massachusetts, 02115, United States of America; 7 Faculty of Health Sciences, Simon Fraser University, Burnaby, BC V5A 1S6, Canada; 8 Departments of Obstetrics and Gynecology, and Preventive Medicine, University of Southern California Los Angeles, Keck School of Medicine, Los Angeles, California, 90033, United States of America; Faculty of Biology, Spain

## Abstract

**Background:**

There are striking differences in breast cancer incidence between Asian and western women. Rates vary substantially within Asia also, with Mongolia's even lower than China's. These profound differences have been speculated to be due in part to diet, mediated by circulating hormone concentrations.

**Methods:**

Sex steroid hormone concentrations were measured in women living in Ulaanbaatar, Mongolia and the United Kingdom (U.K.). Diet was obtained by interview and national survey data. Mean hormone differences were compared by country, and systematic variation by number of days since last menstrual period was modeled and adjusted for age and parity; difference in overall area under the curves was assessed.

**Findings:**

The diet in Mongolia was higher in meat and dairy than in the U.K. Mean testosterone concentrations were 18.5% lower (p<0.0001) while estradiol concentrations were 19.1% higher (p = 0.02) in Mongolian than British women, adjusted for age and parity. Progesterone was almost 50% higher in Mongolian women (p = 0.04), particularly during the follicular phase and early luteal surge. Hormone concentrations generally were similar in Mongolian women born in Ulaanbaatar compared with those born in rural areas, although there was a decreasing progesterone trend by degree of westernization (rural Mongolia; urban Mongolia; U.K.). Mean hormone differences were similar when restricted to parous women, and with further adjustment for body mass index, height, and smoking status.

**Interpretation:**

These data augment accumulating evidence that circulating estrogens are unlikely to explain reduced breast cancer rates in Asia compared with the west, and suggest casting a wider net with respect to biomarkers. Lower testosterone and higher progesterone in Mongolian women raise the possibility that these hormones may be important to consider. In addition, the almost exclusive dietary reliance of Mongolians on meat and dairy argues against beneficial effects of a low-fat diet on circulating hormones explaining international breast cancer differences.

## Introduction

A woman's birth country is one of the strongest established risk factors for breast cancer, with historical pre- and postmenopausal incidence rates in western countries substantially greater than in Asian countries [1] (e.g., estimated age-standardized incidence rates for breast cancer in 2012 were 95 per 100,000 person-years in the United Kingdom (U.K.) and 9.4 per 100,000 in Mongolia) [2], differences for which there is as yet no definitive explanation. Migrant studies indicate that this vast disparity in breast cancer incidence rates is largely a function of environmental factors [3]. While much of the differences could be due to recognized reproductive risk factors, accounting for these still leaves a substantial discrepancy in rates [4]. Another widely-held hypothesis is that lower dietary fat consumption in low-risk compared with high-risk countries explains the reduced risk in the former [5], either directly, or by increasing cumulative circulating estrogens and other sex hormones.

Overlooked in the search for a biologic explanation are the substantial differences in breast cancer incidence rates, as well as life-styles, within Asia, which might be leveraged to help identify the responsible risk factors. We chose to conduct a study in Mongolia, where the reported breast cancer incidence rate is one-third that of China's [6]. We compared selected dietary habits and concentrations of several circulating hormones implicated in breast carcinogenesis between premenopausal women living in Mongolia and the U.K.

## Methods

The study was approved by ethical review boards at Mongolia's Ministry of Health, the National University of Mongolia, the United States (U.S.) National Cancer Institute (National Institutes of Health), the Harvard School of Public Health, and the London School of Hygiene and Tropical Medicine. All participants provided written, informed consent.

### Data collection

In Mongolia, study participants were the mothers of school children attending two primary schools in Ulaanbaator, the capital of Mongolia; one on the outskirts of Ulaanbaatar (to increase the proportion of women who had recently moved from rural areas) and the other in central Ulaanbaatar. Data were collected in the spring of 2009 from mothers who were 45 years of age or younger, and who were not pregnant or breastfeeding. Of the 420 invited women, 100% agreed to participate; 199 from the school in the city's outskirts, and 221 from the school in the city center. Women who were currently using oral contraceptives were excluded, leaving for analysis 137 women born in Ulaanbaatar and 177 women born outside of Ulaanbaatar (born in all but two of the country's 21 other provinces).

Participants were interviewed by trained study staff about demographics, migration and medical history. Dietary intake was based on a short food frequency questionnaire that was developed by one of the authors (GD) which queried participants on their weekly intake of a list of traditional and contemporary Mongolian foods. Weight and height were measured by trained personnel using standardized techniques. Whole blood was collected, and allowed to clot at room temperature. Samples were then centrifuged and sera were stored at −70°C. Participants reported date of first day of last menstrual period and study staff subsequently contacted women to ascertain the first day of the next menstrual period.

A comparison group from a western country was also chosen. These were premenopausal Caucasian women from the U.K. who had been recruited nationally between November 2008 and September 2009 as controls for a case-control study on the genetics of breast cancer (the British Breast Cancer Study) [7], [8]. Each case was asked to nominate an unaffected friend (or a blood-unrelated relative) as a control. These women completed a questionnaire that included self-reported height and weight and had blood samples drawn and processed, with plasma stored at −70°C for hormonal assays. The first day of their last menstrual period and of the next cycle were obtained in the same manner as for the Mongolian women. For the present study, 150 controls were chosen randomly; of these, 129 women were included who were ≤50 years of age and not pregnant, breastfeeding or currently using oral contraceptives. To qualitatively compare dietary patterns with Mongolia, components were estimated from national dietary surveys conducted in the U.K. [9] and China [10].

### Hormone assays

Hormones were measured at the Reproductive Endocrine Research Laboratory at the University of Southern California, Keck School of Medicine under the direct supervision of one of us (FS). Concentrations of estrone, estradiol, testosterone and androstenedione were measured in a single serum or plasma aliquot by radioimmunoassay (RIA) following extraction with organic solvent and separation of the steroids by Celite column partition chromatography, as described previously [11]–[14]. Since progesterone co-elutes in the same fraction as androstenedione on the Celite column, it was processed separately in the same manner as the other steroids. Blinded aliquots of pooled sera and plasma were included with the study samples, constituting 10% of each assay batch. The laboratory technicians also were blinded to country of sample origin. The coefficients of variations (CV) for the blinded serum and plasma samples were 6.2% and 6.5% for androstenedione, 8.2% and 8.1% for testosterone, 6.3% and 8.4% for estradiol, 4.9% and 5.9% for estrone, and 15.0% and 15.6% for progesterone, respectively. Two Mongolian women's values for estrone and estradiol were several SD beyond the next highest values and were excluded from analyses.

As noted, sera were available for the Mongolian women, and plasma for the U.K. comparison group. While plasma and sera hormone assays are accepted as yielding similar values, we assessed this specifically in a methods study by measuring the hormones in paired plasma and serum samples from 10 U.S. women, ages 39 to 42 years, who were not study participants. The laboratory was blinded to whether the sample was plasma or serum. The difference in means, representing combined intraassay error and difference in assay results (sera vs. plasma) was <1% for estradiol, 4.5% for progesterone, 4.3% for androstenedione, 2.7% for testosterone and 7.1% for estrone (**Table S1**), and Pearson's correlations between hormones measured in plasma and serum were, respectively, 0.998, 0.997, 0.997, 0.974 and 0.996. For the statistical analysis, values for each of the hormones were adjusted by adding constants to the serum values for the slight differences in means between plasma and sera as determined in the methods study. Using values with and without this adjustment yielded similar results (data not shown).

### Statistical analysis

The distribution of demographic and lifestyle characteristics for the two populations were compared using chi-square statistics. Tertiles for height and weight were based on the distribution of both populations combined. The menstrual cycle was truncated at 28 days because of small numbers of women with longer cycles (n = 3 in Mongolia; n = 10 in U.K.); results from analyses without truncation were similar (data not shown). Generalized additive models using logarithm transformed hormone values were used to assess mean differences in hormone concentrations between countries, and by migration status within Mongolia. Systematic variation according to the number of days since the first day of the last menstrual period was modeled using quadratic regression splines with evenly spaced knots; the number of segments was selected using the AIC criterion for all women combined without any adjustment for covariate effects [15]. Two spline models were fitted for each hormone, a main effects model with a common set of coefficients for the splined effects of days since last menstrual period, and an interaction model with separate spline coefficients for each population group. The interaction model was used to describe the observed hormone patterns in each group, and the main effects model was used to formally test for mean hormone concentration differences between populations using an F-Test. Both the main effects and interaction models were adjusted for age and parity. Further adjustment for smoking status, height and, body mass index (BMI) was evaluated. The area under the regression spline curve was calculated by summing the estimated spline-smoothed values at days 1–28 and variances were computed using the standard delta method. Statistical significance of the difference in the overall area under the curves, and for the follicular and luteal phases, was estimated from analyses including all days since last menstrual period and for days 1–14 and 15–28, respectively. Regression analyses were repeated excluding the 25% of U.K. women who were nulliparous (all Mongolian women were parous). Least square means for the hormone concentrations by age and parity were calculated using Proc GLM in SAS, v9.2.

## Results

The Mongolian women were younger, and of smaller stature and lower weight than the U.K. women (**Table 1**), although lower BMI in the Mongolians was not statistically significant. All of the Mongolian women (by study design–mothers of school children) and 75% of the U.K. women were parous. Among parous women, about 22% of the U.K. women had one child and 78% had two or more compared with 8.5% and 92% of the Mongolians. Current smoking was higher among Mongolian than U.K. women, although the majority of women in both populations were nonsmokers. Women who migrated to Ulaanbaatar from other Mongolian provinces were slightly older and more likely to be nonsmokers compared with women who were born in Ulaanbaatar.

**Table 1:** Characteristics of Mongolian and U.K. women.

	Born outside UB	Born in UB	p (born outsidevs. in UB)	U.K.	p (U.K. vs. Mongolia combined)
	N = 177	N = 137		N = 129	
Age (years)					
<30	17 (9.7)	22 (16.1))		7 (5.4))	
30–34	58 (33.1)	56 (40.9)		12 (9.3)	
35–39	53 (30.3)	35 (25.6)		40 (31.0)	
40–44	45 (25.7)	24 (17.5)		53 (41.1)	
45–49	2 (1.1)	0 (0.0)	0.006	17 (13.2)	<0.0001
Height (tertiles; cm)					
138.6–156.0	89 (50.3)	53 (38.7)		5 (3.9)	
156.1–162.9	58 (32.8)	60 (43.8)		22 (17.2)	
163.0–183.0	30 (17.0)	24 (17.5)	0.15	101 (78.9)	<0.0001
Weight (tertiles; kg)					
35.8–58.5	71 (40.1)	60 (43.8)		16 (12.5)	
58.6–68.5	62 (35.0)	37 (27.0)		48 (37.5)	
68.6–124.0	44 (24.9)	40 (29.2)	0.94	64 (50.0)	<0.0001
BMI (weight(kg)/height^2^)					
<20	12 (6.8)	10 (7.3)		3 (2.4)	
20–25	79 (44.6)	65 (47.6)		58 (45.7)	
>25	86 (48.6)	62 (45.3)	0.57	66 (52.0)	0.17
Parity					
0	0 (0.0)	0 (0.0)		32 (25.0)	
1	15 (8.5)	21 (15.3)		21 (16.4)	
2+	162 (91.5)	116 (84.7)	0.06	75 (58.6)	<0.0001
Current smoking					
No	162 (91.5)	110 (80.3)		126 (97.7)	
Yes	15 (8.5)	27 (19.7)	0.004	3 (2.3)	<0.0001

UB = Ulaanbaatar.

While the focus of this study was primarily on hormonal profiles, dietary habits ascertained by interview in Mongolia also were compared with those in the U.K. based on previously published national dietary surveys [9] (**Table 2**). The Mongolian diet consists almost exclusively of meat and dairy products at levels greater than those in the U.K. Women born outside Ulaanbaatar in the countryside were more likely to consume the traditional meat and dairy diet [16]. Vegetables and fruits were consumed more often by women born in Ulaanbaatar compared with women born in the countryside, and the former also tended to consume more processed foods and sugar-sweetened drinks. Use of dietary supplements was similar between women born in and outside of Ulaanbaatar. Also for comparison, a published national dietary survey provided data for the Chinese diet of men and women living in urban and rural settings, 18–45 years of age [10]. Compared with the Mongolian diet, the Chinese diet among men and women relies more prominently on vegetables (including soy) and fish. The mean intake per day was approximately 359 g for vegetables (not including potatoes), 29 g for fruit, 92 g for meat, 12 g for milk and milk products and 30 g for fish.

**Table 2:** Components of Mongolian and U.K. diets.

	Mongolia		U.K.	
	Premenopausal women		Women age 19–64	
	Servings/d	g/d*	Servings/d*	g/d
Vegetables (w/o potatoes)	3.58	305	2.18	185
Fruit	1.24	105	1.21	103
Meat	1.64	164	0.88	88
Fish	0.07	7	0.23	23
Milk	1.84	445	0.51	124
yogurt	0.70	170	0.13	32
cheese	0.60	23	0.37	14
Cream, sour cream, butter	1.11	33	0.60	18

*estimated based on the following: For meat and fish 1 serving = 100 g, for vegetables and fruits 1 serving = 85 g, for milk and yogurt 1 serving = 242 g, for butter, cream, and sour cream 1 serving = 30 g and for cheese 1 serving = 30–45 g.

**Figure 1:**
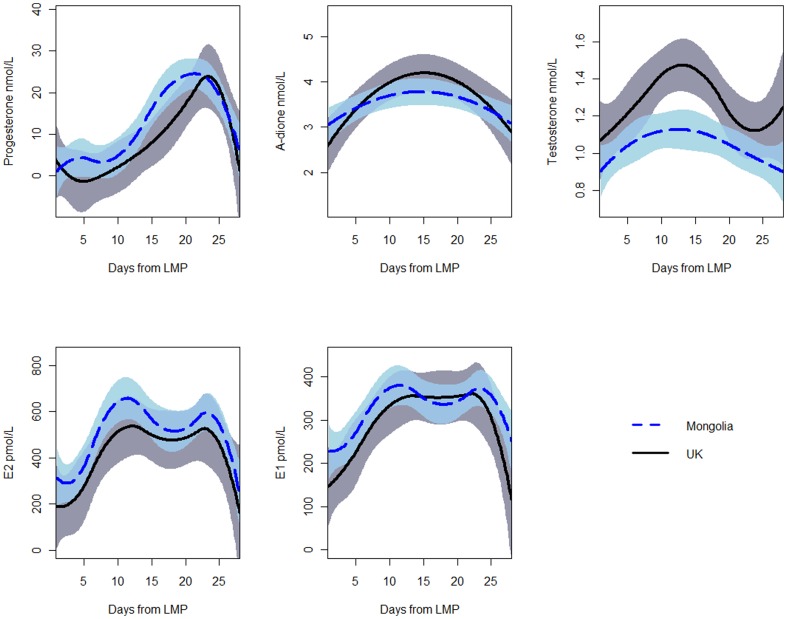
Hormone concentrations in premenopausal Mongolian and U.K. women, adjusted for age and parity. Dashed lines  =  Mongolia; solid lines  = U.K.

With adjustment for age and parity (**Fig. 1** **; Table S2**), mean testosterone concentrations were 18.5% lower in women living in Mongolia than women living in the U.K. (p<0.0001), while mean estradiol (p = 0.02) and progesterone (p = 0.04) concentrations were 19.1% and 48.8% higher, respectively. Androstenedione (p = 0.34; 4.6%) did not differ between groups while higher estrone concentrations in Mongolian women (p = 0.08; 9.3%) did not reach statistical significance. The differences in progesterone, estradiol and estrone concentrations were most pronounced in days 0–14. In fact, the higher mean progesterone concentration in the Mongolian women appeared to be driven also by the earlier and more rapid rise of the luteal surge, with the ultimate peak values being similar between the two groups.

**Figure 2:**
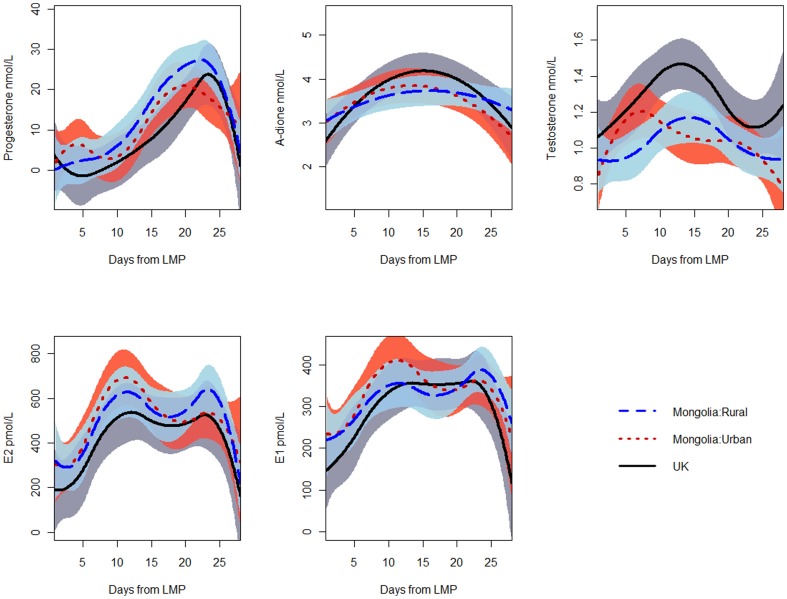
Hormone concentrations in premenopausal Mongolian, born in and outside Ulaanbaatar, and U.K. women, adjusted for age and parity. Dashed lines  =  rural Mongolia; dotted lines  =  urban Mongolia; solid lines  = U.K.

In general, mean hormone concentrations were similar in Mongolian women who were born in Ulaanbaatar compared with those born in the countryside (**Fig. 2**), with the exception of a borderline trend in progesterone with higher values in Mongolian women born in the rural provinces (the lowest progesterone values were in women living in the U.K; p for trend = 0.07). Mean hormone differences noted above were similar in analyses that were restricted to parous women (**S1 Fig.**), or when further adjusted for BMI, height, and smoking status (**S2** **Fig** **.**).

## Discussion

Mongolian women, who have one of the lowest estimated breast cancer incidence rates in the world, have substantially higher premenopausal circulating levels of estradiol and progesterone, and lower levels of testosterone than women in the U.K., who have much higher breast cancer rates. These hormone differences between Mongolian and U.K. women are contrary to what has been assumed and add to the growing evidence that estrogen differences are unlikely to explain the lower breast cancer rates in Asia compared with the west [4]. Instead, they raise interest in the possible etiologic roles for testosterone and progesterone. Also, while our comparisons of dietary habits relied on different methodologies, qualitatively it appears that the Mongolian diet is quite different from other low breast cancer risk Asian populations, being based largely on meat and dairy consumption at levels exceeding that of U.K. women.

While the largest international differences in breast cancer risk relate to the postmenopausal period, there are substantial differences between premenopausal women as well [1]. Furthermore, much of the speculation about the cumulative estrogen dose affects on breast cancer risk pertains to the period before menopause, as cumulative estrogen exposure is largely driven by the premenopausal period because of its higher baseline concentrations. Mongolia's estimates of breast cancer incidence from the International Agency for Research on Cancer (IARC) are based on national data (index of quality  = D5). While some of the lower breast cancer incidence in Mongolia could be due to completeness of the registry compared with other countries, it is unlikely to explain the entire three-fold difference with respect to China. In addition, Mongolia is experiencing dramatic socio-economic changes; as a result, there may be strong cohort effects in breast cancer incidence – i.e. current rates mainly reflect incidence at older ages. The women in the present study were premenopausal and their future breast cancer rates are likely to be higher than the current ones. However; this phenomenon is occurring in other countries in transition as well, such as China. Future differences in breast cancer incidence may be smaller between these countries but are likely to remain.

Other limitations of our study should be mentioned. Our interpretation of hormone differences between countries assumes that the populations sampled are representative of the women living in Mongolia and UK. The population of UK women that we included was born in areas throughout the country. This was also true for the Mongolian population; the women born outside of Ulaanbaatar were from all of the country's provinces except two. This was the best approximation of representativeness that we could achieve. The data we present on hormonal differences, and to the extent possible, dietary differences, are meant to raise hypotheses regarding possible underlying factors that explain the differences in breast cancer incidence between countries. Unfortunately, we did not have exhaustive information on environmental factors in both populations. Factors that possibly differ between countries could include other dietary factors than those presented, breastfeeding practices, stress, immune factors, sanitation, exposure to livestock, and use of tobacco products and alcohol. Also, because we relied on BMI as a measure of adiposity it is possible that adjustment for BMI did not account for differences in fat distribution or other aspects of body fatness.

Speculation on factors responsible for the major differences in breast cancer rates between Asians and Western women have focused primarily on recognized reproductive and anthropometric risk factors, dietary fat intake, and levels of circulating estrogens, with the later acting independently, and/or as a possible biologic mediator of the effects of the other risk factors. While there is evidence that differences in recognized risk factors play a role in explaining the international cancer incidence gaps, they do not explain all of the disparities, and data to support the dietary and hormonal hypotheses are absent or conflicting. Specifically, analytic studies to date in western populations have not established a causal link between dietary fat intake and breast cancer risk [17]. In addition, descriptive studies in Chinese and U.S. women have found lower circulating estrogen levels in the Chinese, however of a magnitude insufficient to explain the differences in risk [4]. This, combined with the results of the current investigation, indicate the need for new hypotheses to explain international differences in breast cancer risk.

Although the differences we observed in hormones in the current study are unlikely to fully explain the large difference in breast cancer incidence rates between low and high risk populations, they provide two possibilities for further consideration – testosterone and progesterone. Increased levels of circulating testosterone have been associated with elevated breast cancer risk in both pre- and post-menopausal women [18], [19]. This has often been dismissed as simply reflecting testosterone's role as an estrogen precursor. It might be prudent to reevaluate the possibility that it plays a more direct role itself. Progesterone has also been a hormone of great interest, but for a variety of mainly practical reasons it has only infrequently been assessed in epidemiologic studies. When it has, no consistent association with breast cancer has emerged. A recent pooled analysis of breast cancer in seven prospective studies found no association with luteal phase concentrations of progesterone [20]. One of the practical problems in measuring progesterone, as well as estrogen, is the measurement error involved in attempting to obtain samples on the same menstrual day for hormones that vary widely over the cycle. Rather than rely on this approach, we sampled women without regard to cycle day and then modeled the entire cycle for the groups we compared. This method has seldom been used by epidemiologists, but we believe it has great advantages both in reducing measurement error, and in allowing evaluation of hormone profiles over the entire cycle. Interestingly, when the pooled analysis above assessed studies that had measured hormones at different times in the menstrual cycle, they did note significantly higher levels of progesterone in controls than cases in the “early luteal phase.” This makes intriguing our observation of progressively higher levels early in the progesterone surge moving from rural Mongolians to urban Mongolians to British women, especially in light of the lower breast cancer rates among the rural compared with urban Mongolians that have been observed [6]. Whether the patterns of progesterone levels in our study reflect greater production by Mongolian women immediately following ovulation, or a shift in ovulation to earlier in the cycle is not clear, and merits further evaluation.

## Panel: Research in Context

### Background

There are striking differences in breast cancer incidence rates between Asian and western women. It is less recognized that incidence varies substantially within Asia, a large geographical area comprised of varying lifestyles and diets, with Mongolian women experiencing one of the lowest rates in the world; one-tenth that of the U.K. The explanation for these profound differences is unknown but has been speculated to be due in part to diet, mediated by circulating hormone concentrations associated with breast cancer risk.

### Interpretation

Our study adds to accumulating evidence that estrogen concentrations do not explain reduced breast cancer rates in Asian countries compared with those in the west, and suggest the utility of casting a wider net with respect to biomarkers. Lower testosterone concentrations and higher progesterone concentrations during the early luteal surge in Mongolian women suggest the possibility that these hormones may be important in the breast cancer protection afforded Asian women. In addition, the heavy dietary reliance of Mongolians on meat and dairy argues against beneficial effects of a low-fat diet on circulating hormones explaining international differences in breast cancer risk.

## Supporting Information

S1 Fig **Hormone concentrations in premenopausal, parous women from Mongolia and the U.K., adjusted for age.** Dashed lines  =  Mongolia; solid lines  = U.K.
(TIF)

S2 Fig **Hormone concentrations in premenopausal Mongolian and U.K. women adjusted for age, parity, smoking status, body mass index and height.** Dashed lines  =  Mongolia; solid lines  = U.K.
(TIF)

S1 Table **Descriptive statistics for reproductive hormones measured in plasma and serum samples from 10 women 39 to 42 years of age.**
(DOCX)

S2 Table **Hormone concentrations in women living in Ulaanbaatar, Mongolia and London, U.K.**
(DOCX)
