# Supplementary figures and images for: The Role of Hormones in the Differences in the Incidence of Breast Cancer between Mongolia and the United Kingdom
Source: PLoS One. 2014 Dec 23;9(12):e114455. doi: 10.1371/journal.pone.0114455 (PMC4275167; doi:10.1371/journal.pone.0114455)

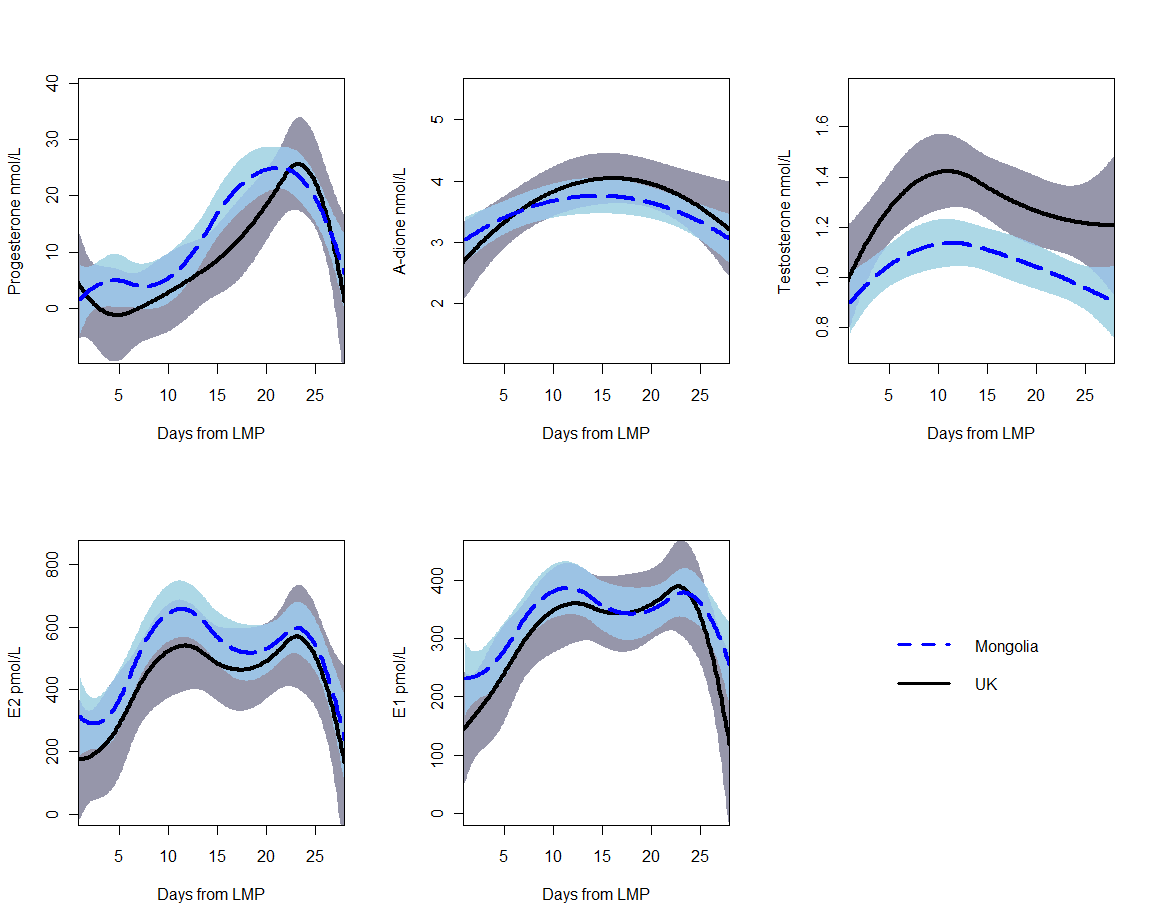

Supplement: S1 Fig — Hormone concentrations in premenopausal, parous women from Mongolia and the U.K., adjusted for age. Dashed lines = Mongolia; solid lines = U.K. (TIF) [file pone.0114455.s001.tif]

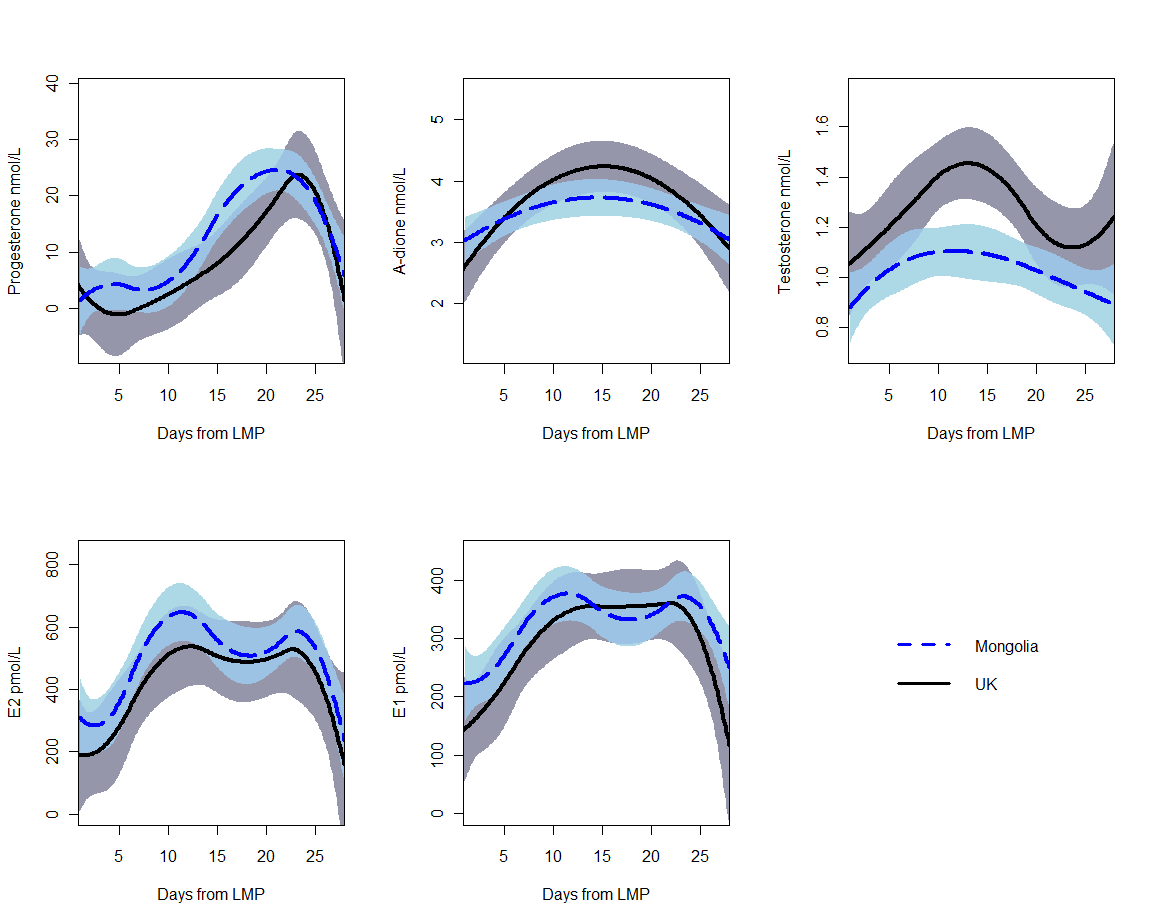

Supplement: S2 Fig — Hormone concentrations in premenopausal Mongolian and U.K. women adjusted for age, parity, smoking status, body mass index and height. Dashed lines = Mongolia; solid lines = U.K. (TIF) [file pone.0114455.s002.tif]
