# Supplementary material for: The Role of Hormones in the Differences in the Incidence of Breast Cancer between Mongolia and the United Kingdom
Source: PLoS One. 2014 Dec 23;9(12):e114455. doi: 10.1371/journal.pone.0114455 (PMC4275167; doi:10.1371/journal.pone.0114455)
Supplement: S1 Table — Descriptive statistics for reproductive hormones measured in plasma and serum samples from 10 women 39 to 42 years of age. (DOCX) [file pone.0114455.s003.docx]

**Table S1. Descriptive statistics for reproductive hormones measured in plasma and serum samples from 10 women 39 to 42 years of age.**

|  | Plasma | | | Serum | | | % difference between plasma and sera | |  |
| --- | --- | --- | --- | --- | --- | --- | --- | --- | --- |
| Hormone | Mean | Median | S.D. | Mean | Median | S.D. | Mean | Median | r |
| Androstenedione nmol/L | 3.28 | 2.72 | 2.44 | 3.14 | 2.72 | 2.23 | 4.3 | 0.38 | 0.997 |
| Testosterone nmol/L | 0.78 | 0.72 | 0.44 | 0.76 | 0.62 | 0.35 | 2.7 | 12.7 | 0.974 |
| Progesterone nmol/L | 11.4 | 1.05 | 25.1 | 11.9 | 0.74 | 27.1 | 4.5 | 28.7 | 0.997 |
| Estrone pmol/L | 466 | 310 | 477 | 434 | 299 | 533 | 7.1 | 3.5 | 0.996 |
| Estradiol pmol/L | 422 | 290 | 404 | 417 | 276 | 389 | <1 | 5.1 | 0.998 |

* per cent difference is calculated based on change from plasma to serum
