# Supplementary material for: The Role of Hormones in the Differences in the Incidence of Breast Cancer between Mongolia and the United Kingdom
Source: PLoS One. 2014 Dec 23;9(12):e114455. doi: 10.1371/journal.pone.0114455 (PMC4275167; doi:10.1371/journal.pone.0114455)
Supplement: S2 Table — Hormone concentrations in women living in Ulaanbaatar, Mongolia and London, U.K. (DOCX) [file pone.0114455.s004.docx]

**Table S2. Hormone concentrations in women living in Ulaanbaatar, Mongolia and London, U.K.**

|  | **Mongolia** | | | **U.K.** | | | **Difference** |
| --- | --- | --- | --- | --- | --- | --- | --- |
| **Hormone** | **N** | **Mean*** | **SD** | **N** | **Mean*** | **SD** | **Per cent** |
| Androstenedione nmol/L | 177 | 3.52 | 0.10 | 129 | 3.70 | 0.17 | 4.6 |
| Testosterone nmol/L | 177 | 1.04 | 0.03 | 129 | 1.29 | 0.05 | 18.5 |
| Progesterone nmol/L | 167 | 11.8 | 1.40 | 121 | 7.92 | 2.13 | 48.8 |
| Estrone pmol/L | 177 | 327 | 14.3 | 129 | 299 | 21.7 | 9.9 |
| Estradiol pmol/L | 177 | 493 | 28.4 | 129 | 414 | 43.2 | 19.1 |

^*^ geometric means are from generalized additive models adjusted for age, parity and the plasma-sera differences determined in the pilot study
